# Antibody Response to *Pneumocystis jirovecii*: Antibody Response to *Pneumocystis jirovecii* Major Surface Glycoprotein

**DOI:** 10.3201/eid1208.060230

**Published:** 2006-08

**Authors:** Kieran R. Daly, Laurence Huang, Alison Morris, Judy Koch, Kristina Crothers, Linda Levin, Shary Eiser, Supriya Satwah, Patrizia Zucchi, Peter D. Walzer

**Affiliations:** * Veterans Affairs Medical Center, Cincinnati, Ohio, USA;; † University of Cincinnati, Cincinnati, Ohio, USA;; ‡ University of California, San Francisco, California, USA;; § University of Southern California, Los Angeles, California, USA;; ¶ Yale University School of Medicine, New Haven, Connecticut, USA;; # University of Pavia, Pavia, Italy

**Keywords:** Human Immunodeficiency Virus (HIV), Acquired Immune Deficiency Syndrome (AIDS), Pneumocystis pneumonia, Pneumocystis jirovecii, Recombinant antigens, Major surface glycoprotein (Msg), Serum antibody responses, Enzyme-linked immunosorbent assay (ELISA), CD4+ cells, First episode pneumocystosis

## Abstract

We conducted a prospective pilot study of the serologic responses to overlapping recombinant fragments of the *Pneumocystis jirovecii* major surface glycoprotein (Msg) in HIV-infected patients with pneumonia due to *P. jirovecii* and other causes. Similar baseline geometric mean antibody levels to the fragments measured by an ELISA were found in both groups. Serum antibodies to MsgC in *P. jirovecii* patients rose to a peak level 3–4 weeks (p<0.001) after recovery from pneumocystosis; baseline CD4+ count >50 cells/μL and first episode of pneumocystosis were the principal host factors associated with this rise (both p<0.001). Thus, MsgC shows promise as a serologic reagent and should be tested further in clinical and epidemiologic studies.

*Pneumocystis jirovecii*, formerly known as *Pneumocystis carinii* special form *hominis* (*1*), is a leading cause of fatal pneumonia in HIV-positive persons and other immunosuppressed patients. Research on *P. jirovecii* has been hampered by the lack of a reliable in vitro culture system, so investigators have developed molecular techniques to characterize isolates. Studies by the Centers for Disease Control and Prevention, San Francisco General Hospital, and other medical centers in the United States that use these techniques have provided epidemiologic insights about *P. jirovecii* patients (*2**,**3*). Reports of *P. jirovecii* colonization detected by molecular probes in persons ranging from healthy young children to adults with chronic lung diseases raise the possibility that this organism may have medical and public health consequences beyond those on the immunocompromised host (*4**,**5*).

Serologic analysis may be useful in epidemiologic studies of *P. jirovecii* infection, especially in light of evidence that antibodies contribute to host defenses against the organism (*6**–**8*). Unfortunately, despite >40 years of investigation, a useful serologic test for *P. jirovecii* is not yet available (*9**,**10*). Antigens have mainly consisted of crude extracts from infected human or rodent lungs. These preparations have shown that antibodies to the organism are highly prevalent in the general population (*4**,**11**,**12*) but have been unable to distinguish present from past infection or colonization from active disease. Specific native *P. jirovecii* antigens have shown more promise as serologic reagents, but they are in short supply (*11**,**13*). This problem has been exacerbated by data about genetic diversity and host specificity of *Pneumocystis*, which have emphasized the importance of matching organisms used in studies with the host from which they have been derived (*1*).

More recent attention has turned toward the use of recombinant *P. jirovecii* antigens to study host immune responses (*14**–**19*). The major surface glycoprotein (Msg or gpA) is highly immunogenic and contains protective B- and T-cell epitopes, and the heavily glycosylated portion of the antigen plays a central role in the interaction of the organism with the host (*6**–**10**,**20*). Msg represents a family of proteins encoded by multiple genes and is thus capable of antigenic variation, which may serve as a mechanism to evade host immune responses. Our strategy has been to use a single Msg isoform that would enable us to begin to understand the host immune response to this complex glycoprotein. We developed 3 overlapping recombinant fragments (MsgA, MsgB, and MsgC), which span the entire length of the *P. jirovecii* Msg, and analyzed their reactivity with serum antibodies in different populations by Western blot (WB) and ELISA (*17**,**18*). A key finding in both studies was that asymptomatic, HIV-positive patients in Cincinnati with a past episode of *Pneumocystis* pneumonia (PCP) had a significantly higher degree of antibody reactivity to MsgC, the carboxyl terminus and most conserved part of the antigen, than patients who had never had the disease.

In this pilot study, we sought to determine whether serum antibody levels to MsgA, MsgB, and MsgC differed in HIV-positive patients with acute pneumonia due to *P. jirovecii* compared to those with pneumonia due to other causes. Further, we asked whether serum antibody levels would rise in these patients during treatment and recovery from pneumocystosis, which Msg fragment could best detect this increase, and whether specific host factors were associated with the antibody rise.

## Materials and Methods

### Patients and Study Design

As standard clinical practice, HIV-positive patients who came to San Francisco General Hospital with respiratory signs and symptoms compatible with pneumocystosis were evaluated by a uniform protocol that has been described previously (*21*). This protocol included obtaining specimens by induced sputum and, if necessary, bronchoscopy with bronchoalveolar lavage. Microscopic examination and cultures were used to establish a specific etiologic diagnosis. Consecutive patients undergoing sputum induction or bronchoscopy to diagnose PCP were enrolled in this study and provided written, informed consent to allow their medical records to be abstracted with a standardized data form. Study investigators classified patients as either PCP positive or PCP negative (controls), according to predetermined definitions that were blinded to serologic results. *Pneumocystis* patients were those patients with a microscopically confirmed diagnosis of *P. jirovecii*; these patients were treated with standard anti-*Pneumocystis* drugs as part of their regular medical care. Control patients were those whose microscopic examinations were negative for *P. jirovecii*, had *Pneumocystis* treatment discontinued, and recovered from acute pneumonia.

The study was conducted during a 4.5-year period (May 2000 through September 2004). During the first half of the study (2000–2002), an acute-phase serum specimen was drawn at the time of hospital admission for pneumonia, and a single convalescent-phase specimen was drawn at different intervals 5–12 weeks later. Preliminary analysis suggested that the *Pneumocystis* patients experienced a rise in mean serum antibody levels, whereas controls did not. Thus, during the later part of the study (2003–2004), additional serial convalescent-phase serum specimens were drawn every 1–2 weeks for 6 weeks from patients with pneumocystosis to measure early changes in antibody levels. Serum specimens were stored at -70°C and shipped to the University of Cincinnati for analysis. University of California San Francisco and University of Cincinnati institutional review boards approved the protocol.

### Analysis of Serum Antibodies

Serum antibody levels to MsgA, MsgB, and MsgC were measured in a blinded manner by an ELISA as previously described (*14**,**17**,**18*). All serum specimens and the standard reference serum were diluted 1:100 and tested in duplicate wells of a 96-well plate against the following reagents: recombinant Msg fragments, *Escherichia coli* extract expressing the pET vector without insert (vector control), tetanus toxoid (TT) (positive control), and phosphate-buffered saline (PBS) without antigen (negative control). As an additional negative control, PBS was substituted for the serum specimen. Plates were washed, horseradish peroxidase (HRP)–labeled goat anti-human immunoglobulin G was added, plates were washed again, and tetramethylbenzidine substrate was added. The reaction was stopped by adding 0.18 mol/L H_2_SO_4_, and the plates were read at a wavelength of 450 nm. The reference serum specimen, which was obtained from a single person and had known reactivity to Msg, was run on each day as another control. HRP-labeled S-protein was used as a positive control and to correct for antigen loading. During the early part of the study, patient and reference serum specimens were tested at 1:100, 1:500 and 1:2,500 dilutions. The best results were obtained with the 1:100 dilution, so this dilution was used for the remainder of the study. The reactivity of each serum specimen to Msg was expressed as the ratio of reactivity to the pET vector: (mean optical density [OD] Msg_test serum_ – mean OD PBS_test serum_) / (mean OD pET_test serum_^__^ mean OD PBS_test serum_).

### Statistical Analysis

Sex and racial distributions of *Pneumocystis* patients and controls were compared by using χ^2^ tests of equality of proportions. Means and standard deviations were calculated to compare the center and spread of age and continuous parameters measured at enrollment (baseline). Mean values for *Pneumocystis* patients and controls were compared and tested for equality by using unpaired *t* tests with adjustment for unequal variances, either on the original or logarithmic scale. Geometric means and geometric standard deviations were calculated when data were approximately lognormally distributed, as judged by Shapiro-Wilks tests and visual assessment. Quantile-quantile plots of serum antibody levels showed that their distributions were approximately lognormal, except for larger numbers of values at the lower quantiles. Pearson product moment correlations among clinical parameters and Msg fragments were obtained. Analyses of trends in mean values of each outcome were performed for all patients enrolled at baseline during either study period and followed up at least once at weeks 1–2, 3–4, or 5–6. Sparse data at later follow-up weeks precluded their inclusion in the regression analyses because of instability of parameter estimates. In addition, the numbers of patients observed at baseline and each follow-up time were too small to provide meaningful inferences from analysis.

Two stratifications of *Pneumocystis* patients were investigated with respect to trends and mean levels of Msg fragments: patients with CD4+ counts above and below the median value (≈50 cells/μL) and patients with and without a history of pneumocystosis. The analysis provided comparisons between means for patient categories at each follow-up week, as well as differences in patterns of change over time between patient groups. We obtained p values that compared mean values between periods and levels of CD4+ count or history of *Pneumocystis* pneumonia by using *t* tests with Tukey's adjustment for multiple comparisons. Patient-specific deviations from group means were included in the analysis as a random effect, which allowed the correlation between repeated measurements on the same patient over time to be included in between- and within-patient variance estimates. A p value <0.05 (2-tailed) was considered significant, unless stated otherwise. Analyses were performed by using the SAS procedure PROC MIXED (SAS for Windows version 9.2, SAS Institute Inc., Cary, NC, USA)

## Results

### Demographic and Clinical Characteristics

No significant differences were seen between the 80 *Pneumocystis* patients and the 41 control patients with other causes of pneumonia in terms of sex, race, or age (Table 1). Baseline serum albumin levels, arterial blood gas measurements, and the proportion of patients who required mechanical ventilation were also similar, which indicates that the overall general health of patients and the severity of pneumonia in these 2 groups were also comparable. In addition, the proportion of patients with prior pneumocystosis was similar in both groups. However, the *Pneumocystis* patients had more advanced or more poorly controlled HIV infection than did the controls. The *P. jirovecii* patients had a significantly lower geometric mean CD4+ count and a significantly higher mean plasma HIV RNA level than did control patients. Patients with pneumocystosis also exhibited a significantly higher mean serum lactate dehydrogenase (LDH) level than did controls; this finding is consistent with the observation that elevated serum LDH is a nonspecific indicator of PCP.

**Table 1:** Demographic characteristics and baseline clinical measurements of patients with *Pneumocystis* pneumonia (PCP) and controls*

Characteristic			PCP (n = 80)	Controls (n = 41)
Demographic				
	Male (%)		86	76
	Race (%)			
		Caucasian	55	39
		African American	29	39
		Other	16	22
	Age at enrollment (y)		41 ± 8	42 ± 8
Clinical				
	CD4 count (cells/μL)†‡		29 ± 3	73 ± 4
	Plasma HIV RNA (copies/mL)†‡		123,130 ± 5	15,582 ± 24
	Albumin (g/L)		3.0 ± 0.5	3.1 ± 0.7
	Prior PCP (%)		24	33
	Pneumonia severity			
	pO_2_ (mm Hg)		65 ± 15.6	73 ± 29
	Aa gradient (mm Hg)		44 ± 13	41 ± 15
	LDH (U/L)†‡		359 ± 2	257 ± 2
	Mechanical ventilation (%)		7	7
Serum antibody levels				
	MsgA†		5.6 ± 4.8	3.7 ± 3.5
	MsgB†		2.9 ± 1.3	1.9 ± 0.9
	MsgC†		4.2 ± 4.3	4.3 ± 3.4

*Unless specified otherwise, values are mean ± standard deviation. pO_2_, oxygen pressure; Aa, alveolar-arterial; LDH, lactate dehydrogenase; Msg, major surface glycoprotein. †Geometric mean ± geometric standard deviation. ‡Significant difference between patient groups for CD4+ count (p<0.01), plasma HIV RNA (p<0.001), LDH (p<0.001).

### Baseline and Sequential Serum Antibody Levels to MsgC

At the time of hospital admission for pneumonia, geometric mean serum antibody levels to MsgC in *Pneumocystis* patients and controls were similar (Table 1). Forty-one of 80 patients with PCP had >1 convalescent-phase serum specimen drawn in the first 6 weeks after hospital admission. The total number of patient visits was 62. Patients observed at weeks 3–4 (n = 19) had a higher mean serum antibody level to MsgC than the average level of all patients at baseline (n = 80) and patients observed at weeks 1–2 (n = 25) and weeks 5–6 (n = 18). Differences were significant, as determined by *t* statistics comparing group means, adjusted for paired comparisons (p<0.01 to p<0.001) (Table 2). Mean antibody levels at subsequent time points were 3.7 at 5–6 weeks, 4.0 at 7–8 weeks, and 3.0 at 9–12 weeks (data not shown).

**Table 2:** Antibody levels to major surface glycoprotein C in *Pneumocystis* pneumonia patients by CD4+ count and week of observation*

Week	All patients		CD4+ <50 cells/μL		CD4+ >50 cells/μL	
	n	Geometric mean (95% CI)	n	Geometric mean (95% CI)	n	Geometric mean (95% CI)
Baseline (0)	80	4.2 (3.1–5.8)	54	4.2 (2.8–6.3)	26	4.4 (2.5–7.6)
1–2	25	8.0 (4.1–15.8)	17	7.3 (3.0–17.8)	8	9.9 (2.8–35.3)
3–4	19	10.4 (4.7–23.1)†	13	6.3 (2.4–16.4)	6	30.4 (7.1–129.5)‡
5–6	18	3.7 (2.0–6.8)	10	5.5 (2.0–15.0)	8	2.3 (1.2–4.4)

*n = no. patients who were observed at the specified follow-up time and at baseline. CI, confidence interval. †p<0.001 vs. week 0, p<0.001 vs. weeks 1–2, and p<0.001 vs. weeks 5–6. ‡p<0.001 vs. week 0, p<0.001 vs. weeks 5–6, p<0.03 vs. weeks 3–4 in CD4+ <50 cells/μL group.

Analysis of serum antibody levels in individual patients showed different patterns of reactivity (Figure). Eleven (58%) of the 19 patients studied at 3–4 weeks had an increase in their antibody levels, ranging from 1.4- to 22-fold above baseline levels. To determine if the rise in serum antibodies in the *Pneumocystis* patients at 3–4 weeks was specific for *P. jirovecii* or part of a broader increase in antibody reactivity, we examined the changes in antibody levels to TT in these 19 patients. The geometric mean antibody levels of 80 U at baseline and 117 U at 3–4 weeks were not significantly different.

**Figure:**
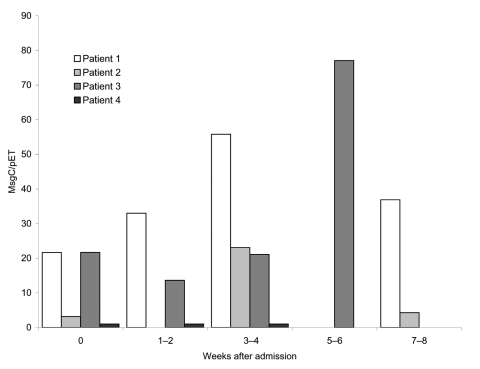
Sequential serum antibody responses to major surface glycoprotein C (MsgC) in HIV-positive patients with pneumocystosis. Patients 1 and 2: antibody levels rose from 21.6 and 3.2 at baseline (point 0) to 55.8 and 23.1 at 3–4 weeks and fell to 36.9 and 4.3, respectively, at 7–8 weeks; patient 3: antibody level showed few changes from baseline until it rose to 77.1 at 5–6 weeks; patient 4: antibody level remained at baseline level of 1.0 throughout.

### Predictors of Serum Antibody Response to MsgC among *Pneumocystis* Patients

We examined clinical characteristics associated with an increase in antibody levels. No relationship was found between antibody level and sex, race, age, HIV viral load, serum albumin level, oxygenation, LDH level, or use of mechanical ventilation. CD4+ cell count (analyzed as CD4+ count <50 cells/μL or >50 cells/μL) was significantly associated with the ability to generate an increase in antibody levels (Table 2). Patients with CD4+ counts <50 cells/μL exhibited no significant changes in antibody levels to MsgC at any time point. In contrast, patients with CD4+ counts >50 cells/μL had a rise in mean antibody level, which peaked at 30.4 at 3–4 weeks and which was significantly higher than antibody levels at baseline (p<0.001) and at 5–6 weeks (p<0.001). The mean antibody level at 3–4 weeks in these patients was also significantly higher than the corresponding level in patients with <50 CD4+ cells (30.4 vs. 6.3, p<0.03).

The lack of a history of pneumocystosis also seemed to influence antibody response but not baseline antibody level. The mean antibody level to MsgC in the 59 patients with first episode of PCP was 4.1; this value was not significantly different from the mean value of 4.2 in the 19 patients with recurrent pneumocystosis (Table 3). Sequential antibody responses were compared in PCP patients whose episode of pneumonia was their first experience with the disease versus those whose pneumonia was a recurrent bout of the disease. Antibody levels in naive patients increased and reached a peak mean value of 17.9 at 3–4 weeks compared to 4.1 at baseline (p<0.001), whereas patients with a history of *P. jirovecii* pneumonia experienced no increase. Because the number of patients was low, we could not analyze the combined effects of PCP history and CD4+ cell count.

**Table 3:** Antibody levels to major surface glycoprotein C in *Pneumocystis* pneumonia (PCP) patients by history of PCP and week of observation*

Week	No history of PCP		History of PCP	
	n	Geometric mean (95% CI)	n	Geometric mean (95% CI)
Baseline (0)	59	4.1 (2.8–6.0)	19	4.2 (2.0–9.0)
1–2	18	9.9 (4.6–21.5)	6	3.9 (0.5–30.8)
3–4	11	17.9 (5.9–53.6)†	6	3.0 (0.7–12.1)
5–6	13	4.4 (2.6–7.4)	4	1‡

*n = no. patients who were observed at the specified follow-up time and at baseline. CI, confidence interval. †p<0.001 versus week 0. ‡Observed values were equal.

### Baseline and Sequential Serum Antibody Levels to MsgA and MsgB

The baseline geometric mean serum antibody level to MsgA of 5.6 in PCP patients was not significantly different from the mean level of 3.7 in the controls (Table 1). Patients with pneumocystosis exhibited a different pattern of antibody response to MsgA than to MsgC in that the mean peak antibody levels to MsgA at 1–2 weeks and 3–4 weeks were similar (Table 4). However, none of the differences in antibody levels at different time points reached significance. In addition, no significant differences were found in the antibody levels at different time points in patients with and without a history of pneumocystosis or in patients with CD4+ cell counts <50 cells/μL or patients with CD4+ counts >50 cells/μL (data not shown).

**Table 4:** Antibody levels to major surface glycoprotein A in *Pneumocystis* pneumonia (PCP) patients by history of PCP and week of observation*

Week	Combined		No history of PCP		History of PCP	
	n	Geometric mean (95% CI)	n	Geometric mean (95% CI)	n	Geometric mean (95% CI)
Baseline (0)	78	5.6 (3.9–7.9)	59	5.8 (3.9–8.7)	19	4.7 (2.1–10.5)
1–2	24	12.0 (6.7–21.4)	18	13.7 (7.0–26.8)	6	7.6 (1.2–47.4)
3–4	17	11.5 (5.2–25.3)	11	13.7 (4.3–43.5)	6	7.3 (1.1–48.9)
5–6	17	5.1 (2.3–11.3)	13	4.0 (1.9–8.2)	4	5.7 (0.2–207.9)

*n = no. patients who were observed at the specified follow-up time and at baseline. CI, confidence interval.

No significant difference in baseline geometric mean serum antibody levels to MsgB was seen in patients with PCP and controls (Table 1). No significant differences were seen in antibody levels related to different time points, CD4+ counts, or history of pneumocystosis (data not shown).

## Discussion

Recombinant antigens derived from *P. jirovecii* have begun to attract attention as possible reagents for analyzing antibodies to *Pneumocystis* in humans (*14**–**19*). We have previously reported that HIV-positive, PCP-positive patients in Cincinnati had a significantly higher frequency and level of serum antibodies to MsgC than did HIV-positive, PCP-negative patients (*17**,**18*); this difference was not found with MsgA or MsgB. These patients were selected on the basis of a history of pneumocystosis and were clinically healthy. The present pilot study has extended these observations to HIV-positive patients hospitalized with acute pneumonia due to *P. jirovecii* and other causes (controls) in San Francisco. The goal of the first part of this study was to determine if baseline antibody levels to the 3 Msg fragments in PCP patients differed from those in the controls. Data showed that the geometric mean antibody levels to MsgC, MsgA, and MsgB were similar in both groups.

In the second part of this study, we analyzed the sequential changes in antibody levels to the 3 Msg fragments in *P. jirovecii* patients after treatment and recovery from pneumocystosis. The results showed a significant rise in mean antibody levels to MsgC that reached a peak at 3–4 weeks. In contrast to MsgC results, no significant changes in antibody levels to MsgA or MsgB occurred at any time point. The pattern of antibody reactivity to these Msg fragments also differed to some degree from the pattern of reactivity to MsgC. Taken together, these data suggest that MsgC is the best Msg fragment to use to analyze antibody responses in this population of HIV-positive patients with active *Pneumocystis* pneumonia.

An antibody rise by 3–4 weeks occurred in 58% of the *Pneumocystis* patients we studied. Of the potential host factors that could affect antibody responses, we were most interested in CD4+ cells, HIV RNA level, and previous history of pneumocystosis. Patients with CD4+ cell counts <50 cells/μL did not mount an antibody response, whereas patients with CD4+ counts >50 cells/μL that peaked 3–4 weeks after diagnosis showed a vigorous antibody response. In contrast to CD4+ count, mean viral load in *P. jirovecii* patients was not associated with an increase in antibody levels. Previous reports have shown that CD4+ cells and HIV itself affect antibody responses in HIV-positive patients, which can be reversed by highly active antiretroviral therapy (HAART) (*22**–**24*). In HIV-positive patients who are severely immunocompromised and have experienced opportunistic infections such as pneumocystosis, however, this immune reconstitution may be incomplete (*25*). Perhaps some who did not respond to treatment fall into this category, but we did not have information about HAART use and immune reconstitution in this cohort.

Our data showed that pneumocystosis patients with or without a previous episode of the disease had similar baseline antibody levels to MsgC; however, patients who experienced their first bout of PCP had better antibody responses after recovery from the disease. Those who were experiencing a recurrent bout of pneumocystosis may have been unable to mount an antibody response to previous episodes and remained at risk. CD4+ cell count or differences in the treatment of pneumocystosis might also play a role in the ability of patients without a previous history of pneumocystosis to mount an antibody response, but our analysis was not powered to analyze multiple factors simultaneously.

Comparison of our results with previous work is complicated by the fact that these earlier studies were performed in the pre-HAART era and involved crude or native antigens. Analysis of whether a detectable antibody response to *Pneumocystis* antigens could develop in HIV-positive patients who recovered from pneumocystosis produced conflicting results (*9**–**11**,**26**–**30*). One report showed a rise in antibodies to native Msg in 43% of HIV-positive patients; host factors such as CD4+ count or pO_2_ could not distinguish responders from nonresponders (*13*). A more recent study that used different recombinant Msg constructs than we used found that HIV-negative, immunocompromised patients who recovered from pneumocystosis had increased antibody levels, but HIV-positive patients who recovered had lower levels and poor antibody responses (*16*). One factor that may contribute to these disparate results is antigenic variation, which involves differences in the Msg constructs themselves. We have developed several variants of our current Msg construct and found that they differ in their ability to distinguish among HIV-positive patients who have and not have had pneumocystosis (unpub. data). PCP patients exhibit greater reactivity with multiple MsgC clones than do patients without PCP or blood donors, but whether the antigens that are recognized are cross-reactive or clone specific is unclear. Further studies to identify broadly reactive MsgC antigens associated with recovery from PCP, as well as proteins (e.g., Kex1) encoded by single-copy genes, would be of interest (*19*).

The sequential serologic results reported here, which were obtained from a limited number of patients, provide the basis for a large, prospective, multisite study of sequential antibody responses to MsgC in HIV-positive patients who have pneumonia caused by *P. jirovecii* and other organisms. Serologic surveys need to be performed in different areas to determine which Msg fragment is the predominant fragment recognized by HIV-positive patients and healthy persons. Standardizing Msg antigen preparations, ELISA conditions, and data analysis would be helpful so that serologic results could be reproduced in different laboratories.

The development of a successful serologic test for *Pneumocystis* infection will have clinical and epidemiologic applications. Serologic tests with MsgC might be used in the diagnosis of pneumonia in situations (e.g., in developing countries) in which a specific cause cannot be established; in cohort studies to investigate the relationship of serum antibody levels and the risk for, and recovery from, *Pneumocystis* pneumonia; in seroepidemiologic surveys and outbreaks of pneumocystosis; and in investigating the pathogenic role for *P. jirovecii* in chronic lung diseases in which colonization of the organism has been detected (*5*).
